# Transcriptomic analysis of Chinese bayberry (*Myrica rubra*) fruit development and ripening using RNA-Seq

**DOI:** 10.1186/1471-2164-13-19

**Published:** 2012-01-13

**Authors:** Chao Feng, Ming Chen, Chang-jie Xu, Lin Bai, Xue-ren Yin, Xian Li, Andrew C Allan, Ian B Ferguson, Kun-song Chen

**Affiliations:** 1 Laboratory of Fruit Quality Biology/The State Agriculture Ministry Laboratory of Horticultural Plant Growth, Development and Quality Improvement, Zhejiang University, Hangzhou 310058, P. R. China; 2 Department of Bioinformatics/The State Key Laboratory of Plant Physiology and Biochemistry, College of Life Sciences, Zhejiang University, Hangzhou 310058, P. R. China; 3 The Horticulture and Food Research Institute of New Zealand, Private Bag 92169, Auckland, New Zealand

## Abstract

**Background:**

Chinese bayberry (*Myrica rubra *Sieb. and Zucc.) is an important subtropical fruit crop and an ideal species for fruit quality research due to the rapid and substantial changes that occur during development and ripening, including changes in fruit color and taste. However, research at the molecular level is limited by a lack of sequence data. The present study was designed to obtain transcript sequence data and examine gene expression in bayberry developing fruit based on RNA-Seq and bioinformatic analysis, to provide a foundation for understanding the molecular mechanisms controlling fruit quality changes during ripening.

**Results:**

RNA-Seq generated 1.92 G raw data, which was then *de novo *assembled into 41,239 UniGenes with a mean length of 531 bp. Approximately 80% of the UniGenes (32,805) were annotated against public protein databases, and coding sequences (CDS) of 31,665 UniGenes were determined. Over 3,600 UniGenes were differentially expressed during fruit ripening, with 826 up-regulated and 1,407 down-regulated. GO comparisons between the UniGenes of these two types and interactive pathways (Ipath) analysis found that energy-related metabolism was enhanced, and catalytic activity was increased. All genes involved in anthocyanin biosynthesis were up-regulated during the fruit ripening processes, concurrent with color change. Important changes in carbohydrate and acid metabolism in the ripening fruit are likely associated with expression of *sucrose phosphate synthase *(*SPS*) and *glutamate decarboxylase *(*GAD*).

**Conclusions:**

Mass sequence data of Chinese bayberry was obtained and the expression profiles were examined during fruit ripening. The UniGenes were annotated, providing a platform for functional genomic research with this species. Using pathway mapping and expression profiles, the molecular mechanisms for changes in fruit color and taste during ripening were examined. This provides a reference for the study of complicated metabolism in non-model perennial species.

## Background

Chinese bayberry (*Myrica rubra *Sieb. and Zucc.) is an economically important subtropical fruit crop native to southern China and other Asian countries [1]. The fruit is popular for its appealing color, delicious taste, and essential micro-nutrients and bioactive constituents such as antioxidants [2] and anti-tumor [3]. The fruit can be eaten fresh, canned, dried, and is widely used in wine-making and juice-making. Chinese bayberry is widely planted not only as a fruit crop, but also for forestry [1]. There is a large germplasm resource for Chinese bayberry and fruit characteristics vary widely among different cultivars [1,4,5]. Moreover, bayberry fruit undergoes rapid changes in pigments, sugars, organic acids and many other quality components during fruit development and ripening, which provides a very useful model for studying fruit quality, particularly properties associated with color, taste and health-associated nutrients.

Despite the economic and ecological importance of Chinese bayberry, there is no genomic resource for this non-model genus, with only 76 nucleotide sequences deposited in GenBank (as at the 30^th ^of November 2011). Current biological studies on this plant, including photosynthesis and stress responses [6], growth regulation [7], and postharvest responses [8-10] have mostly been carried out at the physiological level. To date, the genes involved in anthocyanin biosynthesis as well as a transcription factor gene *MrMYB1 *have been obtained [11] by our group, showing the potential for functional genomic research for this crop. However, a lack of sequence data has become a limitation for extensive and intensive research on this fruit crop.

For woody plants, especially those of high heterozygosity, such as Chinese bayberry, whole genome sequencing requires long-term and expensive investment, and therefore is currently limited to only few species. Instead, it has been more useful to obtain information of UniGenes through transcriptome sequencing [12,13]. The recent RNA-Seq based on NGS (next-generation sequencing) enables studies to be carried out on species without corresponding sequenced genome information as a reference [14]. It has become widely applied to model as well as non-model organisms to obtain mass sequence data for molecular marker development, gene discovery and transcriptional analysis [14-21]. Compared with traditional laboratory methods, RNA-Seq is a high throughput technology, overcoming the weakness of microarrays in exploring unknown genes. Furthermore, it has great advantages in examining transcriptome fine structure, such as detection of allele-specific expression and splice junction variation [22].

In the present work, an RNA-Seq project for Chinese bayberry was initiated (NCBI BioProject Accession: PRJNA77861, http://www.ncbi.nlm.nih.gov/bioproject/77861). Four RNA samples containing various tissues and fruit of different development and ripening stages were sequenced using the latest Illumina deep sequencing technique. The objective of the present study is to gain an understanding of molecular mechanisms of fruit quality changes during ripening and establish a sound foundation for future molecular studies based on high throughput sequence and expression data.

## Results and Discussion

### RNA-Seq

**Figure 1:**
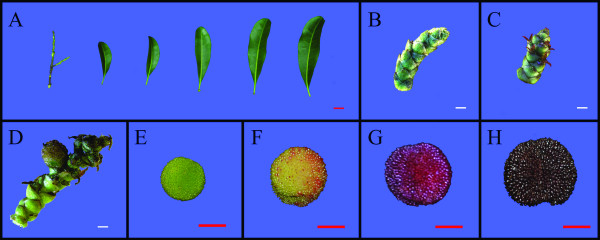
**Tissues of Chinese bayberry cv. Biqi used in deep sequencing**. (A) Stem and leaf, (B) Bud, (C) Flower, (D) young fruit at 15 DAF, (E) young fruit at 45 DAF, (F) breaker stage fruit at 75 DAF, (G) red ripe stage fruit at 80 DAF, (H) dark red ripe stage fruit at 85DAF. Red bar = 1 cm, white bar = 1 mm.

To obtain a general overview of the Chinese bayberry transcriptome, four libraries (MR1, MR2, MR3 and MR4) were designed for RNA-Seq. MR1 was a mixture of equal amounts of RNA from stems and leaves (Figure 1A), buds (Figure 1B), flowers (Figure 1C), and young fruit (Figure 1D, E), while MR2-4 were fruit at breaker stage, red maturity stage, and dark red maturity stage, respectively (Figure 1F, G, H). All samples were taken from a single tree of the cultivar 'Biqi' to provide the same genetic background and thus to make assembly easier. Root RNA was not included in these libraries to avoid potential contamination from mycorrhizal fungi which are not easy to be removed from the root tissue. Each library produced 480 M raw data, from paired-end (PE) reads with a single read length about 90 bp and a Q20 percentage (percentage of sequences with sequencing error rate lower than 1%) over 93% (Table 1). This is higher than the 88% for similar work from tea [16], likely to be due to the development of the sequencing technology. All these data showed that the throughput and sequencing quality was high enough for further analysis.

**Table 1:** Throughput and quality of RNA-Seq of Chinese bayberry cv. Biqi libraries

Libraries	Total Reads	Total nucleotides (nt)	Q20 percentage^a^	N percentage^b^	GC percentage
MR1	5.3 M	480 M	93.2%	0.0%	50.1%
MR2	5.3 M	480 M	94.0%	0.0%	49.5%
MR3	5.3 M	480 M	93.8%	0.0%	49.8%
MR4	5.3 M	480 M	94.3%	0.0%	49.2%

^a ^Q20 percentage indicates the percentage of sequences with sequencing error rate lower than 1%, ^b ^N percentage indicates the percentage of nucleotides which could not be sequenced.

In previous works, take Illumina platform as an example, PE reads of 75 bp or 90 bp were *de novo *assembled to obtain transcriptome reference, and then 21 bp-tags or 50 bp single-end (SE) reads, from other runs of deep sequencing, were mapped back to the reference for expression annotation in different transcripts [19,20]. In this study, however, a novel experimental design was applied. Four RNA samples, each ligated with a different adapter, were sequenced altogether in a same run. The data produced from the mixed samples were used to construct the whole transcriptome file, and meanwhile, they were used as the reference and combine with data from each separate sample to do further gene expression analysis. In addition to being less costly, longer reads were easier and more probably to be mapped into correct transcript sequences than shorter ones, especially for the higher plant species which contain more gene family members. This strategy can be generally ideal for species without a good reference.

### *De novo *assembly

Short reads from four libraries were assembled into 199,438, 139,918, 137,722 and 174,012 contigs with a mean length of 147, 159, 156 and 148 bp, respectively. These were assembled into Scaffolds and UniGenes, taking the distance of PE reads into account. MR1 contained approximately 38,000 UniGenes, while the other libraries had around 30,000, with a mean length of UniGenes from each library ranging from 406 to 437 bp (Additional File 1). All these sequences were assembled to give 41,239 non-redundant UniGenes with a mean length of 531 bp. A N50 length of 708 bp, i.e., half of the assembled bases were incorporated into UniGenes with a length at least 708 bp, was obtained, with one third (13,115 UniGenes) having a length over 500 bp (Additional File 2A). The average gap percentage (ratio of number of 'N' to UniGene length) was 1.4%. 76.6% of the UniGenes had no gap, and less than 2% of the UniGenes had gap over 20% (Additional File 2B). The mean depth of UniGenes was 52.9, while 44.3% and 5.5% of UniGenes had the depth over 10 and 100, respectively (Additional File 2C).

### Functional annotation

**Figure 2:**
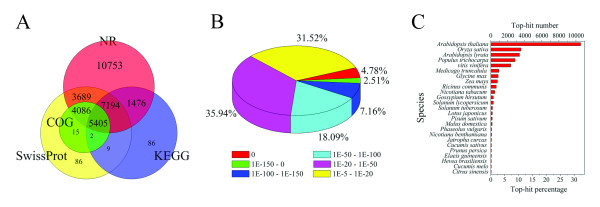
**Characteristics of homology search of Chinese bayberry UniGenes**. (A) Venn diagram of number of UniGenes annotated by BLASTx with an E-value threshold of 10^-5 ^against protein databases. The numbers in the circles indicate the number of UniGenes annotated by single or multiple databases, (B) E-value distribution of the top BLASTx hits against the nr database for each UniGene, (C) Number and percentage of UniGenes matching the 25 top species using BLASTx in the nr database.

Approximately 80% of UniGenes (32,805) were annotated by BLASTx, with a threshold of 10^-5^, to four public databases (NCBI non-redundant (nr) database, the Swiss-Prot protein database, the Kyoto Encyclopedia of Genes and Genomes (KEGG) database, and the Clusters of Orthologous Groups of proteins (COG) database). Among them, 32,603 UniGenes could be annotated to the nr database, while 5,404 UniGenes could be annotated to all the databases (Figure 2A). Based on nr annotation and the E-value distribution, 68.5% of the mapped sequences showed strong homology (E-value < 10^-20^), and 32.5% were very strong homology (E-value < 10^-50^) to available plant sequences (Figure 2B). The 25 top-hit species based on nr annotation are shown in Figure 2C. Nearly 70% of UniGenes can be annotated with sequences from 5 top-hit species, i.e., *Arabidopsis thaliana, Oryza sativa, Arabidopsis lyrata, Populus trichocarpa*, and *Vitis vinifera*. Over 7,000 UniGenes were classed into three gene ontology categories: cellular component, biological process, and molecular function. Under the cellular component category, large numbers of UniGenes were categorized as cell and organelle. For the biological process category, metabolic process (2,839 UniGenes, 38.1%) and cellular process (2,665 UniGenes, 35.8%) represented the major proportion. Under the molecular function category, binding (3,751 UniGenes, 42.6%) and catalytic activity (3,175 UniGenes, 38.4%) were the top two most abundant sub-categories (Additional File 3).

There were 14,172 UniGenes mapped into 125 KEGG pathways. The maps with highest UniGene representation were Plant-pathogen interaction (Ko04626, 1,010 UniGenes, 7.13%), followed by Spliceosome (Ko03040, 897 UniGenes, 6.33%) and Ribosome (Ko03010, 424 UniGenes, 2.99%). The pathways with highest representation were purine metabolism (Ko00230, UniGenes, 2.99%), starch and sucrose metabolism (Ko00500, 389 UniGenes, 2.75%) and pyrimidine metabolism (Ko00240, 366 UniGenes, 2.58%), respectively (Additional File 4). From these pathways, information on Chinese bayberry metabolism can be obtained. As an example, biosynthesis of ascorbic acid (AsA, vitamin C), an important component of bayberry fruit, was examined. There are four biosynthetic pathways for AsA in plants, the Smirnoff-Wheeler (S-W), galactonate, glucose, and myo-inositol pathways [23-26]. In Chinese bayberry, the sequences for all 9 genes in the S-W pathway were assembled; however, this was not the case for the other three pathways (Additional File 5). Therefore, it is reasonable to conclude that S-W pathway is the main pathway for the biosynthesis of AsA in Chinese bayberry, unlike that in kiwifruit, which possesses both S-W and the myo-inositol pathways [13] and in strawberry, where the galactonate pathway was firstly discovered [24]. Such annotations provide a good platform for further research into understanding metabolic pathways in this species. In contrast, the KO (KEGG Orthology) ids were used in Interactive pathways (Ipath) analysis and were helpful for the study of fruit quality related metabolism (see below).

Although only 62.4% of all annotated UniGenes were found in the SwissProt database (considerably less than in the nr database), the functional annotation provided by this database may be more reliable due to manually reviewed annotation. It was noteworthy that all the 9,509 UniGenes annotated against COG were included in SwissProt annotated UniGenes (Figure 2A). The COG database represented major phylogenetic lineages of Chinese bayberry, as shown in Additional File 6. This data would be useful in researching protein classification and evolution rates. The fact that 15.3% (2,586) of 16,916 UniGenes share homology with signal transduction (group T) or transcription factors (group K), which is higher than those in tomato [27], confirms that bayberry fruit development and ripening requires complex regulatory processes.

Through BLASTx against the databases mentioned above, the direction and region of CDS (coding sequences) were extracted from the sequences. 31,665 CDS were translated into peptide sequences, over 10% of which (3,714) had a length over 300 AA, with the 34 longest UniGenes having a length over 1000 AA. The detailed length distribution is shown in Additional File 2D.

### Expression profiling during ripening

**Figure 3:**
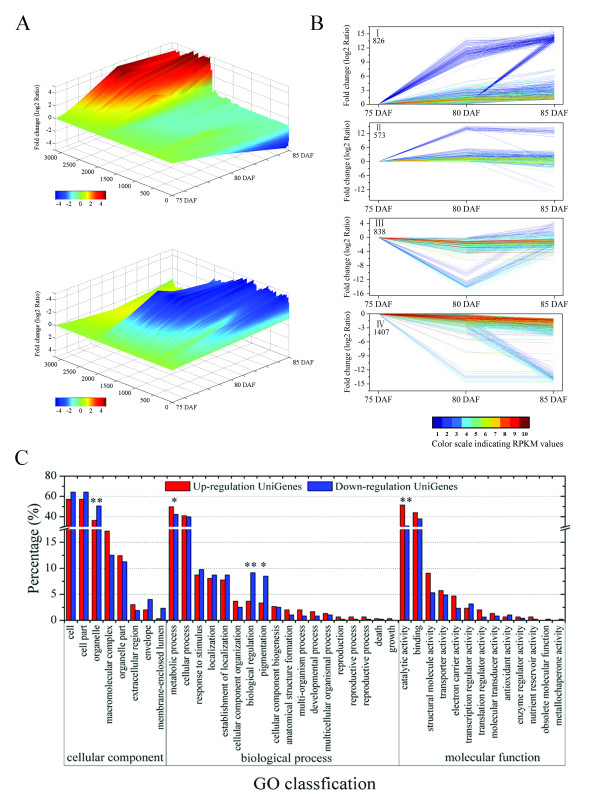
**UniGene expression profiles during bayberry fruit ripening**. (A) Overall expression profiles for the UniGenes expressed in fruit libraries of three different maturity stages, (B) Four expression profiles are shown, with I and IV indicating UniGenes with up-regulated and down-regulated expression, respectively, and II and III indicating those with irregular expression. The lines with 10 different colors from blue to red show the absolute expression magnitude at 75 DAF, with the RPKM values 0-10, 10-20, 20-40, 40-80, 80-160, 160-320, 320-640, 640-1280, 1280-2560, and over 2560 represented by colors 1 to 10, respectively, (C) GO classification for up-regulated and down-regulated UniGenes, with * and ** indicating significant difference at 5% and 1%, respectively.

The RPKM method, first proposed by Mortazavi in 2008 [28], is widely used in expression annotation of RNA-Seq data. We therefore used this method as a means to calculate the expression of UniGenes in Chinese bayberry. 37,000 UniGenes were expressed during fruit ripening, with 1,947, 2,801 and 554 showing differential expression between MR2 and MR3, MR2 and MR4, MR3 and MR4, respectively. When mixed together, 3,644 UniGenes were differentially expressed during fruit ripening and hence were further analysed. For a global view of expression patterns, the expression level of 3,152 UniGenes which changed less than 32 fold (2^5^), was visualized in 3 dimensional space (Figure 3A). This gives an overall understanding about the expression changes of UniGenes. Moreover, 4 groups were defined according to their expression profiles, containing 826, 573, 838 and 1,407 UniGenes, respectively. Group I was defined as up-regulated, group IV as down-regulated, and groups II and III had irregular expression patterns (Figure 3B). The lowly expressed UniGenes at the start point could change over 1,000 fold. Ten groups of UniGenes with the similar expression trends were identified and shown in Additional File 7. And the detailed co-expression relationships of the top 12 related UniGenes listed in Additional File 8.

The proportions and comparisons between up-regulated and down-regulated UniGenes were summarized in three main functional categories (Figure 3C). Metabolism and catalytic activity were major responsive classes, where the number of up-regulated UniGenes was significantly higher than those down-regulated. This suggests that metabolic processes are enhanced and catalytic activity increases during fruit ripening.

### Metabolic pathway analysis during fruit ripening

**Figure 4:**
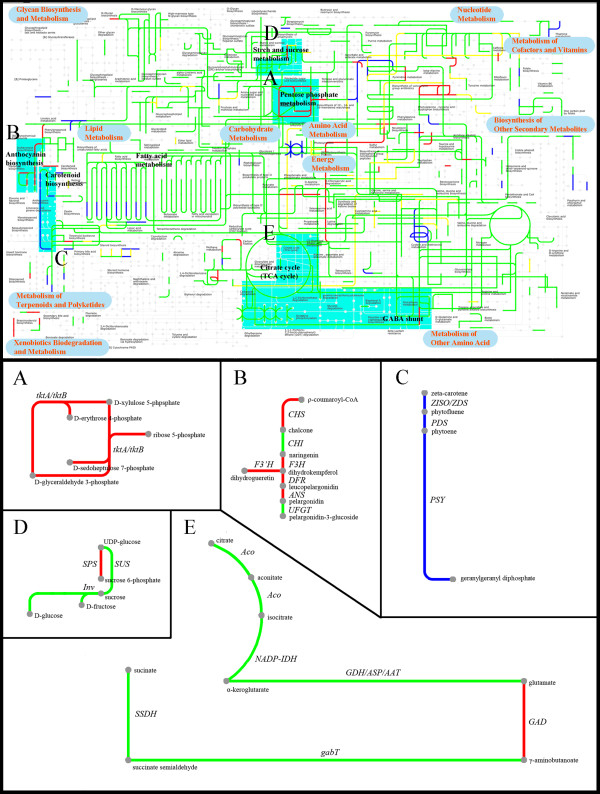
**Interactive pathways analysis during bayberry fruit ripening**. The green, the red, the blue and the yellow lines indicate genes with non-significant expression change, up-regulated, down-regulated, and irregularly regulated, respectively. The areas with sky blue background indicate the metabolic pathways related to fruit color, sugar and organic acids. (A) Pentose phosphate metabolism, (B) Anthocyanin biosynthesis, (C) The upstream part of carotenoid biosynthesis, (D) Sucrose biosynthesis, (E) GABA shunt.

To provide a global view of Chinese bayberry metabolism, 2,369 genes with different KO ids were submitted for analysis via the on-line Interactive Pathways (ipath) explorer v2 and mapped to 1,173 pathways (Figure 4). The dynamic change and absolute expression magnitudes during fruit ripening were shown in Additional File 9. The lines shown in Figure 4 indicated genes that mapped to pathways including metabolism of lipids, carbohydrates, amino acids, nucleotides and energy metabolism. Green lines indicated that the expression of most members in a specific gene family did not differ significantly over fruit ripening. Some pathways (indicated as red lines), such as pentose phosphate metabolism (Figure 4A) which generates NADPH for increasing the metabolic rate during ripening, showed enhancement, which was in accord with the result of GO comparisons (Figure 3C). The expression of many elements encoding anthocyanin biosynthesis was also increased (Figure 4B). However, the genes encoding the first three steps of the carotenoid biosynthesis pathway from geranylgeranyl pyrophosphate (GGPP) to zeta-carotene decreased (Figure 4C), which was consistent with the absence of carotenoids in fully ripe fruit. Expression of *SPS *(*sucrose phosphate synthase*) and *GAD *(*glutamate decarboxylase*) increased (Figure 4D, E), while the genes involved in the TCA cycle showed no significant change in expression.

Interactive pathways (Ipath) is an open-access online tool that integrates 123 KEGG maps from 183 species [29]. It has been used in association with RNA-Seq transcriptomics [30], for example, in the analysis of *Solanum *glandular trichomes [31], and global metabolic map analysis of horned beetles [32]. In the present study, the Ipath application provided an interactive metabolic net associated with gene expression changes during fruit ripening in Chinese bayberry. A focus was then made on two important fruit quality traits: color and taste.

### Genes related to color development

**Figure 5:**
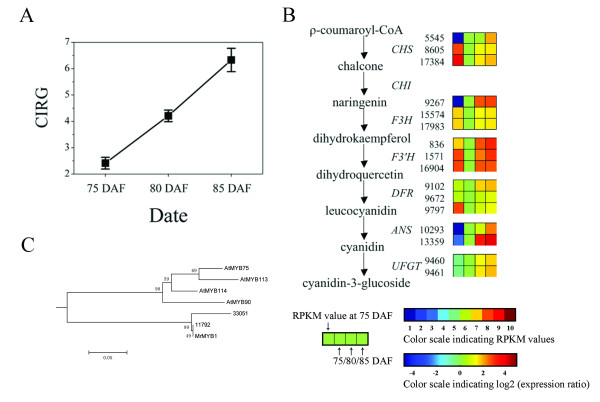
**Schematic of physiological and metabolic data related to bayberry fruit color during ripening**. (A) Changes in CIRG values during fruit ripening, (B) anthocyanin biosynthesis pathway. Enzyme names, UniGene ids and expression patterns are indicated on the right of each step. The expression patterns of each UniGenes are shown by 4 grids, with the left one representing the RPKM value at 75 DAF, and the second to fourth ones from the left to the right representing the relative log_2 _(expression ratio) at 75, 80, 85 DAF, respectively. The grids with 10 different colors from blue to red show the absolute expression magnitude at 75 DAF, with the RPKM values 0-10, 10-20, 20-40, 40-80, 80-160, 160-320, 320-640, 640-1280, 1280-2560, and over 2560 represented by colors 1 to 10, respectively, (C) An anthocyanin-related branch of the phylogenetic tree comparing Chinese bayberry R2R3-MYBs amino acid sequences with all MYBs from *Arabidopsis thaliana*.

During the later stages of development, Chinese bayberry (cv. Biqi) undergoes a rapid change in color, from green to dark red, due to the degradation of chlorophylls and the biosynthesis of anthocyanins, mainly cyanidin-3-glucoside. Comparing three ripening stages, the CIRG value, which matched visual perception of color differences of Chinese bayberry [8], increased from 2.41 to 6.33 (Figure 5A), reflecting the increase in intensity of color as fruit ripens (Figure 1F, G, H). Previous literatures suggested that the fruit anthocyanin content at full maturity is well correlated with the cumulative expression of anthocyanin biosynthetic genes in many crops, including grape [33], apple [34], litchi [35], and Chinese bayberry [11]. In this work, UniGenes participating in the anthocyanin biosynthetic pathway were selected and studied in detail, and it was observed that expression of 16 Unigenes (GenBank accession no.: JR053922 to JR053937) were up-regulated (Table 2, Figure 5B and Additional File 10). All the genes encoding anthocyanin biosynthesis, except for *chalcone isomerase *(*CHI*), showed significantly up-regulated expression, and the expression of one UniGene encoding *CHI *also increased 1.8 fold between 75 DAF and 85 DAF. In addition, Ipath analysis also showed that 5 out of 7 steps for the biosynthesis of anthocyanin were up-regulated during fruit ripening (Figure 4B). These results coincided with the q-PCR results from previous work [11]. A co-expression network for the 16 up-regulated UniGenes was built, and 153 UniGenes with similar expression trends were examined (Additional File 11). A sub-set of these may have functions related to anthocyanin biosynthesis, which could be of value to be further evaluated.

**Table 2:** Candidate genes involved in bayberry fruit quality related metabolism

Pathway	Gene	Enzyme	KO id (EC-No.)	No. All^a^	No. Change^b^	No. Up^c^	No. Down^d^
Genes related to color development							

	*CHS*	Chalcone synthase	K00660 (2.3.1.74)	6	4	3	1
	*CHI*	Chalcone isomerase	K01859 (5.5.1.6)	2	0	0	0
	*F3H*	Flavanone 3-hydroxylase	K00475 (1.14.11.9)	7	3	3	0
	*F3'H*	Flavanone 3'-hydroxylase	K05280 (1.14.13.21)	5	3	3	0
Anthocyanin biosynthesis	*DFR*	Dihydroflavonol 4-reductase	K00091/K13082 (1.1.1.219)	8	3	3	0
	*ANS*	Anthocyanidin synthase	K05277 (1.14.11.19)	9	4	2	1
	*UFGT*	UDP-glucose:flvonoid 3-*O*-glucosyltransfersae	K12930 (2.4.1.115)	7	3	2	1

Genes related to taste development							

Sucrose metabolism	*SPS*	Sucrose phosphate synthase	K00696 (2.4.1.14)	15	5	5	0
	*SUS*	Sucrose synthase	K00695 (2.4.1.13)	3	0	0	0
& transport	*Inv*	Invertase	K01193 (3.2.1.26)	8	3	2	1
	
	*SUT*	Sucrose transporter	/^e^	10	0	0	0

	*Aco*	Aconitase	K01681 (4.2.1.3)	7	0	0	0
	*NADP-IDH*	NADP-isocitrate dehydrogenase	K00031 (1.1.1.42)	9	1	0	0
	*GDH*	Glutamate dehydrogenase	K00261 (1.4.1.3) K00262 (1.4.1.4)	8	1	0	0
	*AST*	Aspartate aminotransferase	K00813 (2.6.1.1)	7	0	0	0
GABA shunt	*AAT*	Alanine aminotransferase	K00814 (2.6.1.2)	3	0	0	0
& citrate	*GAD*	Glutamate decarboxylase	K01580 (4.1.1.15)	3	2	2	0
transport	*gabT*	gamma-aminobutyrate-aminotransferase	K00823 (2.6.1.19)	4	1	0	1
	*SSDH*	Succinate-semialdehyde dehydrogenase	K00135 (1.2.1.16)	2	0	0	0
	*GS*	Glutamine synthetase	K01915 (6.3.1.2)	9	1	1	0
	
	*V-ATPase*	Vacuolar H^+^-ATPase	\^f ^(3.6.3.14)	41	3	3	0
	*Cit*	Citrate/H^+ ^symporter	/^e^	1	0	0	0

^a ^No. All indicates the total number of UniGenes analysed, ^b ^No. Change indicates the number of UniGenes with expression significantly changed during fruit ripening, ^c ^No. Up indicates the number of UniGenes with expression significantly up-regulated during fruit ripening,

^d ^No. Down indicates the number of UniGenes with expression significantly down-regulated during fruit ripening, ^e ^/ indicates that this protein is not an enzyme, ^f ^\ indicates omission of numbers of Ko id because different subunits of V-ATPase have different Ko ids.

The correlated expression of anthocyanin biosynthetic genes with color change during ripening is consistent with coordinated regulation by a MYB transcription factor, as reported in many other fruits [36]. Of the 55 R2R3-MYBs present in the Chinese bayberry data-set, two UniGenes (11,792 and 33,051) were clustered together with *AtMYB75 *and *AtMYB90*, which were identified as transcription factors related to anthocyanin biosynthesis (Figure 5C). The UniGene 11792 had 100% similarity in amino acid sequence to MrMYB1, which has been reported to control bayberry fruit anthocyanin biosynthesis [11]. However, the UniGene 33051 showed a premature translation termination codon, which would result in a peptide of only 73 amino acids, rather than around 250 amino acids for MrMYB1, and a very low expression magnitude, and is therefore unlikely to have a role in regulating anthocyanin biosynthesis. Overexpression of MYB transcription factors can lead to increased anthocyanin production in transgenic tomato, apple and strawberry [36-38], and similar phenotypic changes were observed in tobacco and *Arabidopsis *transformed with *MrMYB1 *(data not shown).

### Genes related to taste quality

**Figure 6:**
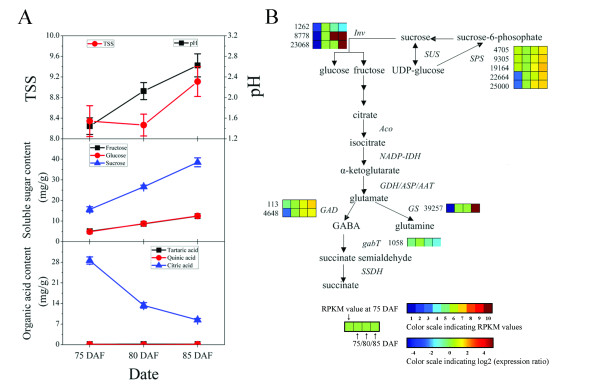
**Schematic of physiological and metabolic data related to bayberry fruit taste during ripening**. (A) Changes in TSS, pH, content of soluble sugars, and content of organic acids, (B) Sucrose metabolism and organic acid degradation through the GABA shunt. Enzyme names, UniGene ids and expression patterns are indicated at the side of each step. The expression patterns of each UniGene are shown by 4 grids, with the left one representing the RPKM value at 75 DAF, and the second to fourth ones from the left to the right representing the relative log2 (expression ratio) at 75, 80, 85 DAF, respectively. The grids with 10 different colors from blue to red show the absolute expression magnitude at 75 DAF, with the RPKM values 0-10, 10-20, 20-40, 40-80, 80-160, 160-320, 320-640, 640-1280, 1280-2560, and over 2560 represented by color 1 to 10, respectively.

**Figure 7:**
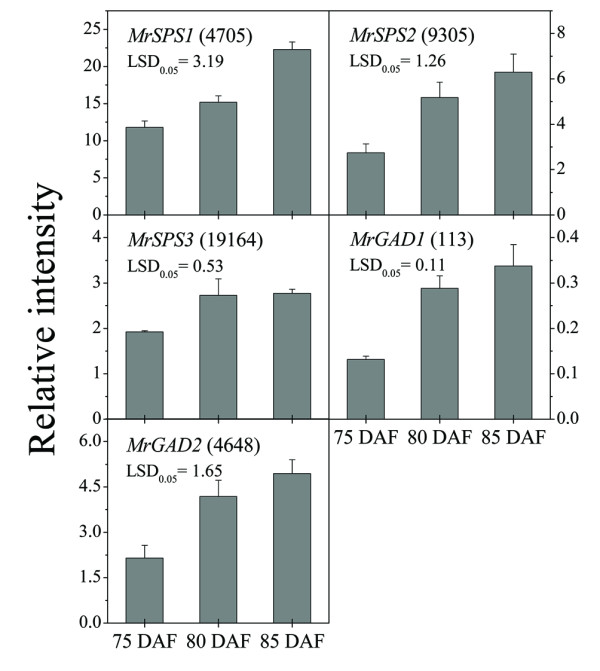
**Expression of *SPS *and *GAD *in bayberry fruit ripening**. Error bars on each column indicate SEs from three replicates.

Sugar and acidity provide the most important contributions to taste. During fruit ripening, Chinese bayberry fruit became sweeter and less acetous. Total soluble solids (TSS), an indicator of the degree of sweetness, increased from 8.3 to 9.1. Sucrose, which accounts for approximately 60% of the total soluble sugars, rapidly increased from 15.7 mg/g to 38.1 mg/g (Figure 6A). 26 UniGenes involved in sucrose metabolism were identified (Table 2), and the expression of five UniGenes encoding *sucrose phosphate synthase *(*SPS*) (GenBank accession no. JR053941 to JR053943) increased rapidly (Table 2, Figure 6B and Additional File 10) during fruit ripening, which was consistent with q-PCR results (Figure 7). The expression of *sucrose synthase *(*SUS*) did not show significant change. All the UniGenes annotated as invertase, which converts sucrose into glucose and fructose, showed low expression magnitude, in spite of the fact that two UniGenes were up-regulated and one down-regulated (GenBank accession no.: JR053938 to JR053940, Table 2, Figure 6B and Additional File 10). When considered together with the Ipath data (Figure 4D), it was concluded that *SPS *was likely to be the key gene controlling sucrose accumulation in Chinese bayberry fruit, as what observed in citrus [39], sugarcane [40], and banana [41]. Furthermore, ten candidate UniGenes for sucrose transporters (SUT) were examined (Additional File 12), but none showed significant expression change during fruit ripening (Table 2).

The pH value of bayberry fruit juice increased from 1.4 to 2.5 during fruit ripening. The content of citric acid, the main organic acid, which accounts for over 95% of total organic acids, decreased from 28.6 mg/g to 8.4 mg/g (Figure 6A). The genes encoding the TCA cycle showed no significant change in expression during fruit ripening (Figure 4). As to the GABA shunt, gene expression of all three UniGenes encoding *glutamate decarboxylase *(*GAD*) were increased, two of which (GenBank accession no.: JR053944 and JR053945) were up-regulated significantly (Table 2, Figure 6B and Additional File 10), which coincided with the q-PCR results (Figure 7). It has been reported that *GAD *activity participated in regulating cytosolic pH [42]. Therefore, the up-regulation of *GAD *expression may be one of the key factors in citric acid degradation. Meanwhile, one UniGene encoding *glutamine synthase *(*GS*) (GenBank accession no.: JR053946) increased significantly (Table 2, Figure 6B and Additional File 10), with the expression magnitude of this gene always high (Additional File 9). Therefore, glutamate might be utilized by other pathways, causing the loss of accumulation of citric acid. A similar viewpoint was proposed by Cercos in citrus [43].

It is widely known that citrate can be transported, mediated by a vacuolar H^+^-ATPase or a Citrate/H^+ ^symporter, between the vacuole and cytoplasm to maintain homeostasis [44]. It was suggested that a large influx of protons is required and that this is mediated by the vacuolar H^+^-ATPase [45,46], a multi-heteromeric complex of at least 11 different subunits [47]. During fruit ripening, the expression of vacuolar H^+^-ATPase A subunit was increased in tomato [48] and grape [49], while a number of other subunits are still largely uncharacterized at the molecular level. In the present study, 41 UniGenes encoding vacuolar H^+^-ATPase subunits, with one encoding subunit A, were found. Three UniGenes, but none of them encoding subunit A, showed up-regulated expression (Table 2). Further more detailed work is needed to elucidate whether and how vacuolar H^+^-ATPase participates in regulation of organic acid accumulation in Chinese bayberry fruit. On the other hand, citrate may also be removed from the vacuole via a Citrate/H^+ ^symporter. The expression of a citrus symporter (*CsCit1*) decreased in line with the reduction in acidity during fruit ripening [50]. In Chinese bayberry, however, only one UniGene encoding a Citrate/H^+ ^symporter was detected but this showed no change in gene expression during fruit ripening (Table 2).

### Future perspectives

Transcription factors play important roles in regulation of plant growth, including fruit development and ripening. Additional File 5 shows 1,508 UniGenes classified as transcription factors based on annotation by the COG database, accounting for 8.9% of the annotated UniGenes. Through BLASTx to TFs downloaded from PlantTFDB with an E value threshold of 10^-20^, 2,284 UniGenes encoding transcription factors from 57 families were obtained. In future work these transcription factors could be used to build a network between TFs and their target genes, based on their co-expression relationships.

Chinese bayberry has rich germplasm resources, with variable fruit characteristics in size, color, taste, texture and bioactive components [1-5,8-11]. With the data provided by this study, critical genes involved in development and regulation of these fruit quality attributes can be characterized and the mechanisms elucidated at the molecular level, which can further contribute to understanding of fruit biology as well as exploring manipulations for fruit production and quality improvement. Currently, we are carrying out further RNA-Seq of fruit from various cultivars with different contents of organic acids. Based on this information and related physiological data, we hope to build a regulatory network of citric acid metabolism in Chinese bayberry fruit.

## Conclusions

Based on mass sequence data of Chinese bayberry obtained by RNA-Seq, many UniGenes were identified and annotated, which provides an excellent platform for future genetic and functional genomic research. Genes related to fruit quality and their expression profiles through three fruit ripening stages were analysed further. This offered new insights into the molecular mechanisms underlying Chinese bayberry fruit characteristics. The up-regulated UniGenes encoding anthocyanin biosynthesis and *MrMYB1 *played roles in accumulation of anthocyanin and the development of the deep dark-red color. The up-regulated expression of *SPS *could be associated with the increase in sweetness, while *GAD *may participate in accumulation and loss of organic acids during fruit ripening. The study provides a platform for further functional genomic research on this fruit crop, and a reference for study of complicated metabolism in non-model perennial species.

## Methods

### Plant material

All samples were collected from the same Chinese bayberry (*Myrica rubra *Sieb. and Zucc. cv. Biqi) tree growing in Yuyao County, Zhejiang Province, China. Figure 1 shows different tissues and fruit of three different ripening stages sampled. All samples were transported to the laboratory within 4 hours after picking and fruit were screened for uniform size and absence of mechanical damage, before freezing in liquid nitrogen and storing at -80°C.

### RNA extraction, library construction and RNA-Seq

Total RNA was extracted as described previously [51] and treated with DNase (Takara, China). The MR1 library was constructed by mixing equal quantities of RNA from stems and leaves, flowers, buds, and young fruit. The MR2-4 libraries consisted of separate RNA samples from fruit of three different ripening stages, i.e., 75 DAF (the breaker stage fruit), 80 DAF (red ripe stage fruit), and 85 DAF (dark red ripe stage fruit). The fruit RNA used for RNA-Seq was extracted from mixed samples of 15 fruit picked from the same tree. Enrichment of mRNA, fragment interruption, addition of adapters, size selection and PCR amplification and RNA-Seq were performed by staff at Beijing Genome Institute (BGI) (Shenzhen, China). mRNA was isolated with Oligo(dT) cellulose, and then broken into short fragments. Taking these short fragments as templates, first-strand cDNA and second-strand cDNA were synthesized. Sequencing adapters were ligated to short fragments after purifying with QiaQuick PCR extraction kit, which were used to distinguish different sequencing samples. Fragments with lengths ranging from 200 to 700 bp were then separated by agarose gel electrophoresis and selected for PCR amplification as sequencing templates. Finally, the four libraries were sequenced using Illumina HiSeq™ 2000.

### *De novo *assembly

The raw reads were first filtered by removing the adapter sequences and low quality sequences, which included the reads with N percentage (i.e., the percentage of nucleotides in read which could not be sequenced) over 5% and ones containing more than 20% nucleotides in read with Q-value ≤ 10. The Q-value represents the sequencing quality of related nucleotides. Clean reads were used in *de novo *assembly and read-mapping to the transcriptome. RNA-Seq data was *de novo *assembled using the SOAPdenovo assembly program (version 1.04) [52] at the parameters of "-K 29 -M 2 -L 50" by BGI. The meaning and selection principles of the parameters were available on Internet (http://soap.genomics.org.cn/soapdenovo.html). For each library, short reads were first assembled into longer but gapless contigs. Then the reads were mapped back to contigs, taking the distance of PE reads as frame, unknown sequences were replaced with 'N's, and then Scaffolds were made. The gaps of Scaffolds were filled by PE reads in order to get the sequence with least 'N's, and thus sequence of the UniGene produced. After that, UniGenes from four libraries were further spliced and assembled to obtain non-redundant UniGenes by TGICL with the minimum overlap length of 100 bp [53], and this was used for further analysis in this study.

### Functional annotation

Function of UniGenes was annotated by BLASTxing with E-value threshold of 10^-5 ^to protein databases including the NCBI non-redundant (nr) database, the Swiss-Prot protein database, the Kyoto Encyclopedia of Genes and Genomes (KEGG) database [54], and the Clusters of Orthologous Groups of proteins (COG) database. The proteins with the highest sequence similarity were retrieved for analysis. KEGG produced annotation of metabolic pathways, while COG matched each annotated sequences to an ancient conserved domain, and represented major phylogenetic lineages of Chinese bayberry. Based on nr annotation, 25 top-hit species were identified and Gene ontology (GO) classification was obtained by WEGO [55] (http://wego.genomics.org.cn/cgi-bin/wego/index.pl) via GO id annotated by Blast2GO (Version 2.3.4) [56]. Moreover, coding sequence (CDS) regions and direction were determined based on those of similar sequences from other plants. Phylogenetic analysis, based on amino acid sequences of *MrMYB1 *(GenBank accession no. GQ340767), R2R3-MYBs projected in this work and R2R3-MYBs in *Arabidopsis *were performed by MEGA (version 5.02) [57] using Neighbor-joining methods with 1000 bootstrap replicates.

### Expression annotation

An alignment package, SOAPaligner (Version 2.20) was used to map reads back to the transcriptome at the parameters of "-m 0 -x 1000 -s 40 -l 35 -v 3 -r 2". The meaning and selection principles of the parameters were available on Internet (http://soap.genomics.org.cn/soapaligner.html). Then the number of mapped clean reads for each UniGene was counted and then normalized into RPKM value (Reads Per kb per Million reads), which was widely used to calculate the UniGene expression [28]. P value was used to identify genes expressed differentially between each samples following the formula below via our PERL program (Additional File 13). N1 represented the total clean reads in Sample 1, while the total clean reads in Sample 2 were noted as N2, and gene A contained × and y clean reads mapped to Sample 1 and 2, respectively [58].

$$2\overset{\text{i}=\text{y}}{\underset{\text{i}=0}{\sum }}\text{p}\left(\text{i}|\text{x}\right)\left(\text{if}\overset{\text{i}=\text{y}}{\underset{\text{i}=0}{\sum }}\text{p}\left(\text{i}|\text{x}\right)\leq 0.5\right)\;\text{or}\;2\times \left(1-\overset{\text{i}=\text{y}}{\underset{i=0}{\sum }}\text{p}\left(\text{i}|\text{x}\right)\right)\left(\text{if}\overset{\text{i}=\text{y}}{\underset{\text{i}=0}{\sum }}\text{p}\left(\text{i}|\text{x}\right)>0.5\right)$$

$$\text{p}\left(\text{y}|\text{x}\right)={\left(\frac{{\text{N}}_{2}}{{\text{N}}_{1}}\right)}^{\text{y}}\frac{\left(\text{x}+\text{y}\right)\text{!}}{\text{x!y!}{\left(1+\frac{{\text{N}}_{2}}{{\text{N}}_{1}}\right)}^{\left(\text{x}+\text{y}+1\right)}}$$

FDR (false discovery rate) was applied to identify the threshold of the P Value in multiple tests and analyses [59], and this value was calculated via SAS (version 9.1.3). In our work, the differentially expressed UniGenes between each of two samples were screened with the threshold of FDR < 0.001 and the absolute value of log_2_Ratio ≥ 1 [21]. Overall expression patterns of UniGenes, excluding those with no significant expression changes as determined by FDR analysis or with changes over 32 fold, were visualized by 3D using MATLAB (version 7.0) after clustering via cluster 3.0. Detailed expression profiles I (up-regulation), II (irregularly regulated), III (irregularly regulated), IV (down-regulation) were distinguished for the differentially expressed UniGenes via value of log_2 _(RPKM _80 DAF _/RPKM _75 DAF_) and log_2 _(RPKM _85 DAF _/RPKM _80 DAF_), where I = (the former one ≥ 0 and the latter one ≥ 0), II = (the former one ≥ 0 and the latter one ≤ 0), III = (the former one ≤ 0 and the latter one ≥ 0), IV = (the former one ≤ 0 and the latter one ≤ 0). Furthermore, the GO classifications were compared between up-regulation and down-regulation UniGenes using WEGO [55], with * and ** indicating significant difference at 5% and 1%, respectively.

### Co-expression analysis

Based on RPKM value of differentially expressed UniGenes, clustering analysis was performed via MultiExperiment Viewer (MeV) (version 4.6.2) [60] using the algorithm of "K Means Clustering". Co-expressed gene networks were built using the following steps: Firstly, UniGenes with RPKM value equal to 0 were filtered, then PCC (Pearson's correlation coefficients) value of a pair of UniGene expression patterns, considering sample redundancy, were calculated following the formula of the online help page (http://atted.jp/help/coex_cal.shtml) and further transformed into Mutual Rank (MR) with the method described (http://atted.jp/help/mr.shtml) [61]. After that, co-expressed gene relationships were generated from the 12 most strongly correlated genes for each gene. Finally, the branch of the network related with UniGenes in the anthocyanin biosynthesis pathway was examined using Cytoscape (version 2.8.2) by drawing a line between co-expressed genes.

### Metabolic pathways analysis

Interactive Pathways (ipath) analysis was carried out via interactive pathways explorer (version 2.0) (http://pathways.embl.de/). Through KO (KEGG Orthology) id, the expression of a specific gene family was summed from all family members encoding the gene. To understand the dynamic changes and absolute expression magnitude during fruit ripening, ten different colors were applied to indicate different RPKM values of UniGenes. For obtaining accurate relative expression magnitude, another ipath figure was generated, where the standard of change was FDR < 0.001 and the absolute value of log_2_Ratio ≥ 1 as mentioned above [21]. Metabolic pathways related with fruit color, sugar and organic acids were produced manually. The sequences of related UniGenes (described in Figure 5B and Figure 6B) were deposited in the Transcriptome Shotgun Assembly Sequence Database (TSA) at NCBI with accession number from JR053922 to JR053947. Expression and detailed functional annotation are shown in Additional File 10.

### RNA extraction, first strand cDNA synthesis and q-PCR analysis

Total RNA used for q-PCR analysis was extracted from fruit of three ripening stages (75 DAF, 80 DAF and 85 DAF), using three biological replicates of 15 fruit. After RNA extraction and DNase treatment described above, the first-strand cDNA was synthesized from 1.0 μg DNA-free RNA using Revert Aid™ First Strand cDNA Synthesis Kit (Fermentas, USA) according to the manufacturer's protocol. The cDNA was diluted tenfold and used as the template for q-PCR.

The q-PCR mixture (20 μl per volume) comprised of 10 μl SYBR^® ^Premix Ex TaqTM (Takara, China), 0.4 μl of each primer (10 μM) (Table 3), 2 μl of cDNA, and 7.2 μl of RNase-free water. The reactions were performed on a LightCycler 1.5 instrument (Roche, USA) by the two-step method, which was initiated by 30s at 95°C, then followed by 45 cycles of 95°C for 5 s, 60°C for 20 s, and completed with a melting curve analysis program. The specificity of q-PCR primers was confirmed by melting curve and sequencing of q-PCR products. The expression was calculated by 2^-ΔCt ^and normalized to the actin gene (*MrACT*, GenBank accession no. GQ340770) [11], and LSDs (α = 0.05) were calculated for mean separations using the Data Processing System (DPS, version 3.01). Three UniGenes encoding *SPS *and two UniGenes encoding *GAD *(Table 3) were selected for q-PCR analysis, in consideration of their higher expression abundance and longer sequence length (Additional File 10).

**Table 3:** Primers for q-PCR analysis

Genes	**GenBank accession no**.	Forward primer (5' to 3')	Reverse primer (5' to 3')	Product size(bp)
*MrSPS1 *(4705)	JR053938	TGTGATCCTGAAGGGTGTGG	CGAAGCTCGAATGTCGTTGC	139

*MrSPS*2 (9305)	JR053939	TTGATGGAAAGAACAGAGCT	TTTCAAGAAGATCTGAGGAT	192

*MrSPS3 *(19164)	JR053940	AGGGAGAGGAAGCTCAGCAT	GAGCAGAAATATCACTAACC	132

*MrGAD*1 (113)	JR053944	CCTGTATTTGGGCTTGGTA	ATTTCTCATTTCGAGTTCC	182

*MrGAD2 *(4648)	JR053945	GTTAAGAGGTTTGTCCTGT	GGTTAGGTAAACTAGTCCAA	160

### Color, TSS (total soluble solids) and pH Measurement

Fruit surface color at different ripening stages was measured using a Hunter Lab Mini Scan XE Plus colorimeter (Hunter Associates Laboratory, Inc., USA). The CIE L*a*b* color scale was adopted, and the raw data as L*, a*, b* were obtained. The CIRG, a comprehensive indicator of the color index of red grapes, was calculated according to CIRG = (180-*H*)/(L*+*C*), while C = (a*^2 ^+b*^2^)^0.5 ^and H = arctan(b*/a*) [8,62]. Four random measurements were made for each fruit and a mean value was obtained from the measurements of ten fruit.

After color measurement, the fruit was used to measure the TSS and pH with refractometer PR101-α (Atago, Japan) and pHTestr 30 (Eutech, USA) following the manufacturers' protocols. Two measurements were made for each fruit and a mean was obtained from the measurements of ten fruit.

### HPLC measurements

The extraction of sugars and organic acids was performed according to previous methods [8]. Briefly, three grams of the sample was ground into fine powder in liquid N_2_, and then homogenized in 6.0 ml of ethanol (80%) solution, shaken for 10 min at 35°C and centrifuged at 10,000 rpm for 10 min. The supernatant was collected and the precipitate homogenized again with ethanol (80%). This procedure was repeated, the supernatants were made up to a constant volume of 25 ml. The extract (1 ml) was dried into powder by a Termovap Sample Concentrator (Eyela, Japan), and then the volume was made up to 500 μl with distilled water, which was used for sugar and organic acid analysis.

Soluble sugars were analysed by HPLC (Beckman, USA) following Koch's method [63]. Acetonitrile: water (80: 20) was used as the mobile phase at a flow rate of 1.0 ml/min. A NH_2 _(4.6 mm × 250 mm) column (GL Sciences Inc., Japan) and a refractive index detector RI-1530 (Jasco, Japan) were used.

Organic acids were analysed by HPLC (Beckman, USA) following the method of Shiomi [64]. 50 mM (NH_4_)_2_HPO_4 _(pH = 2.7) was used as the mobile phase at flow rate of 0.5 ml/min, using a Beckman ODS C18 (4.6 mm × 250 mm) column (Beckman, USA) and a 166 UV-vis detector (Beckman, USA).

## List of abbreviations

AAT: Alanine aminotransferase; Aco: Aconitase; ANS: Anthocyanidin synthase; AsA: Ascorbic acid, vitamin C; AST: Aspartate aminotransferase; BGI: Beijing Genome Institute; BLAST: Basic local alignment search tool; CDS: Coding sequence; CHI: Chalcone isomerase; CHS: Chalcone synthase; CIRG: Color index of red grapes; COG: The clusters of orthologous groups of proteins database; DAF: Days after flowering; DFR: Dihydroflavonol 4-reductase; EST: Expressed sequence tag; F3H: Flavanone 3-hydroxylase; F3'H: Flavanone 3'-hydroxylase; FDR: False discovery rate; GABA: Gamma aminobutyrate; GabT: Gamma-aminobutyrate transaminase; GAD: Glutamate decarboxylase; GDH: Glutamate dehydrogenase; GGPP: Geranylgeranyl pyrophosphate; GS: Glutamine synthase; Inv: Invertase; Ipath: Interactive pathways; KEGG: The Kyoto encyclopedia of genes and genomes database; KO: KEGG Orthology ids; NADP-IDH: NADP-isocitrate dehydrogenase; NCBI: National center for biotechnology information; NGS: Next-generation sequencing; nr: NCBI non-redundant database; PE: Paired-end; PERL: Practical extraction and report language; q-PCR: Real-time quantitative PCR; RNA-Seq: RNA-sequencing; RPKM: Reads per kb per million reads; SE: Single-end; SOAP: Short oligonucleotide analysis package; SPS: Sucrose phosphate synthase; SSDH: Succinate-semialdehyde dehydrogenase; SUS: Sucrose synthase; SUT: Sucrose transporters; S-W: Smirnoff-Wheeler pathway; TCA: Tricarboxylic acid cycle; TSS: Total soluble solids; UFGT: UDP-glucose: flavonoid 3-*O*-glucosyltransfersae; UTR: Untranslated region.

## Authors' contributions

CF carried out the experiment, analyzed the data and drafted the manuscript. MC designed the data analysis and participated in writing of the manuscript. CX contributed to the research design and participated in writing the manuscript. LB participated in data analysis and wrote the PERL program. XY supported technically and participated in data analysis. XL and ACA contributed to data analysis and reviewed the manuscript. IBF contributed to the research design and reviewed the manuscript. KC initiated the project, designed the research framework and coordinated the study. All authors read and approved the final manuscript.

## Supplementary Material

Additional File 1 **Length and gap distribution of Contigs, Scaffolds and UniGenes from each library of Chinese bayberry cv. Biqi**. Click here for file

Additional File 2 **Overview of the length, gap and depth distribution of Chinese bayberry UniGenes**. (A) Length distribution, (B) Gap percentage (ratio of number of 'N' to UniGene length) distribution, (C) Depth distribution, (D) Length distribution of deduced amino acid sequences. Click here for file

Additional File 3 **GO classification of Chinese bayberry UniGenes**. Click here for file

Additional File 4 **Pathway annotation of Chinese bayberry UniGenes**. Click here for file

Additional File 5 **Ascorbic acid biosynthesis pathway in Chinese bayberry**. The number of UniGenes is shown besides each step. Click here for file

Additional File 6 **COG classification of Chinese bayberry UniGenes**. Click here for file

Additional File 7 **Clustering analysis of differentially expressed UniGenes**. Click here for file

Additional File 8 **Co-expression relationships of differentially expressed UniGenes**. Top 12 related UniGenes are shown in this Table. Click here for file

Additional File 9 **Dynamic Interactive pathway analysis on bayberry fruit during ripening**. This flash file shows the dynamic changes in absolute expression magnitude of specific gene families from 75 DAF to 80 DAF and finally 85 DAF. The expression was summed from all members encoding a gene from the same family, identified through KO id. The lines with 10 different colors from blue to red show the absolute expression magnitude, with the RPKM values 0-10, 10-20, 20-40, 40-80, 80-160, 160-320, 320-640, 640-1280, 1280-2560, and over 2560 represented by color 1 to 10, respectively. Click here for file

Additional File 10 **Expression annotation and functional annotation of UniGenes shown in Figure 5B and Figure 6B**. Click here for file

Additional File 11 **The branch of co-expression network related with UniGenes in the anthocyanin biosynthesis pathway**. The UniGenes encoding anthocyanin biosynthesis enzymes and related co-expressed UniGenes are indicated with yellow and pink red circles, respectively, and a line is drawn between co-expressed genes. Click here for file

Additional File 12 **Top BLAST results of Chinese bayberry sucrose transport protein (SUT) to *Arabidopsis *and other plants based on amino sequence**. Click here for file

Additional File 13 **A PERL program applied in the p-value calculation**. Click here for file
