# Supplementary figures and images for: Transcriptomic analysis of Chinese bayberry (Myrica rubra) fruit development and ripening using RNA-Seq
Source: BMC Genomics. 2012 Jan 13;13:19. doi: 10.1186/1471-2164-13-19 (PMC3398333; doi:10.1186/1471-2164-13-19)

A

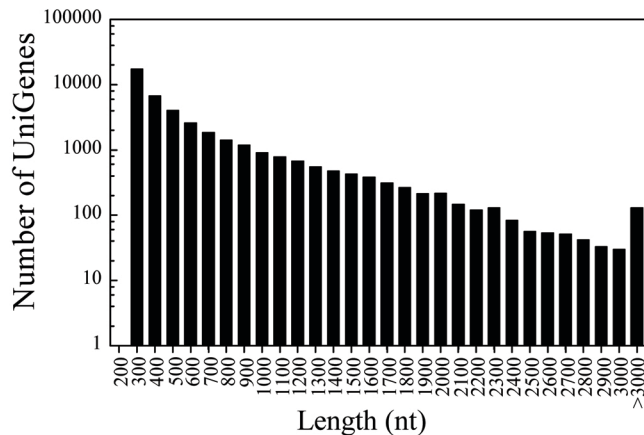

B

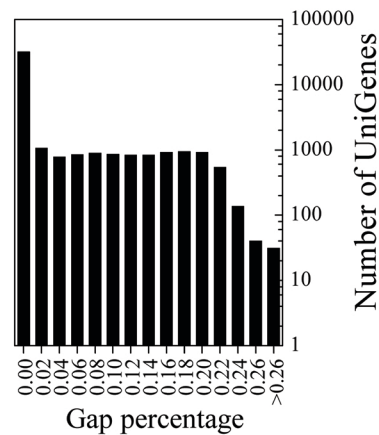

C

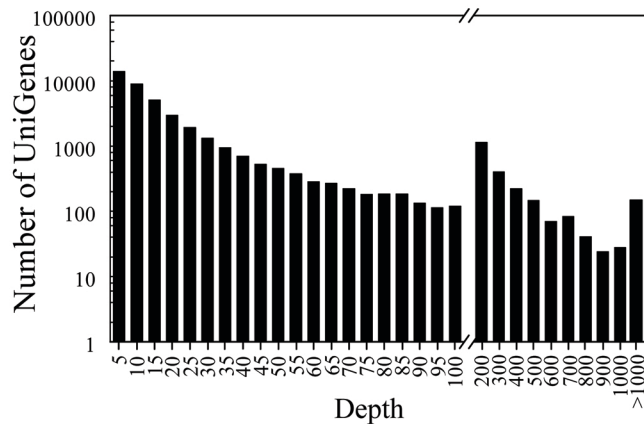

D

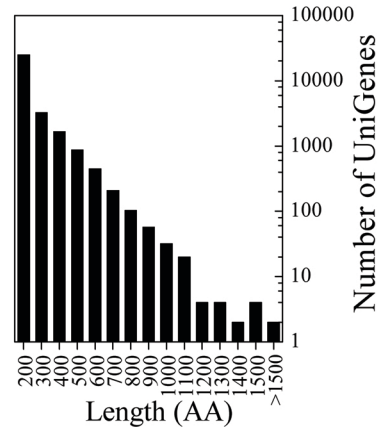

Supplement: Additional File 2 — Overview of the length, gap and depth distribution of Chinese bayberry UniGenes. (A) Length distribution, (B) Gap percentage (ratio of number of 'N' to UniGene length) distribution, (C) Depth distribution, (D) Length distribution of deduced amino acid sequences. [file 1471-2164-13-19-S2.PDF]

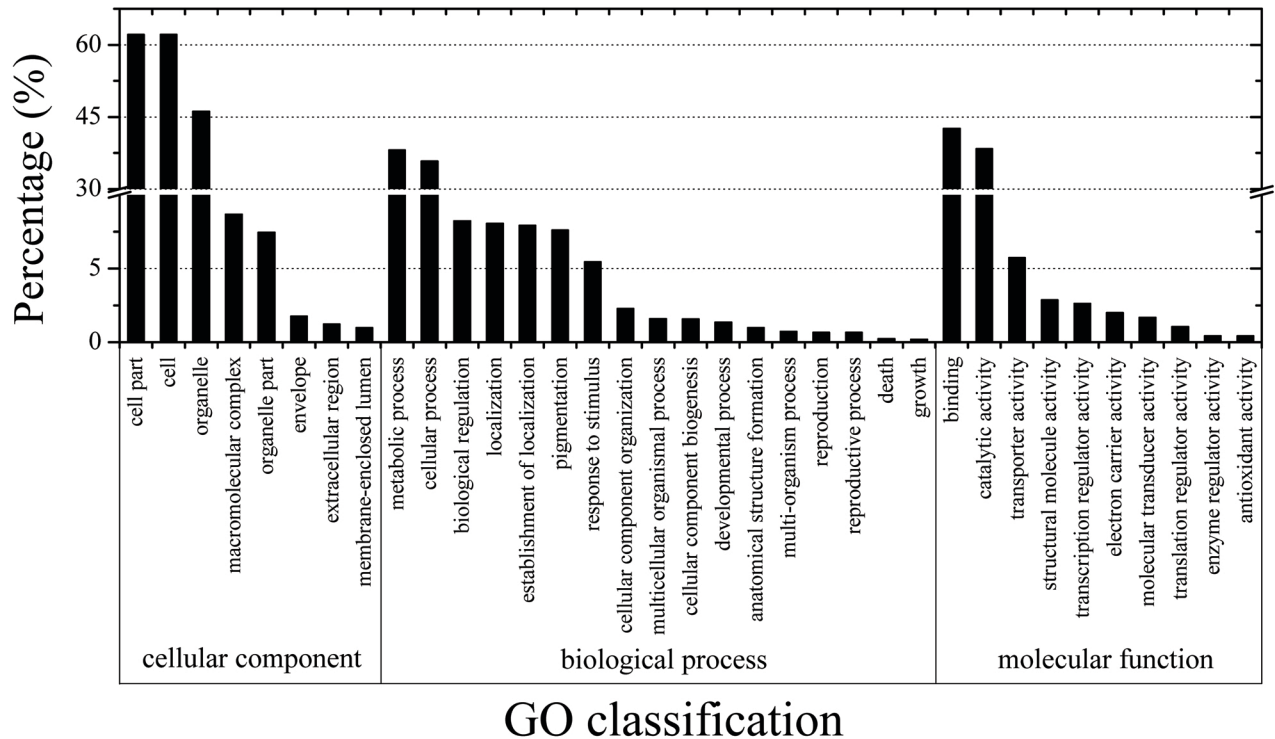

Supplement: Additional File 3 — GO classification of Chinese bayberry UniGenes. [file 1471-2164-13-19-S3.PDF]

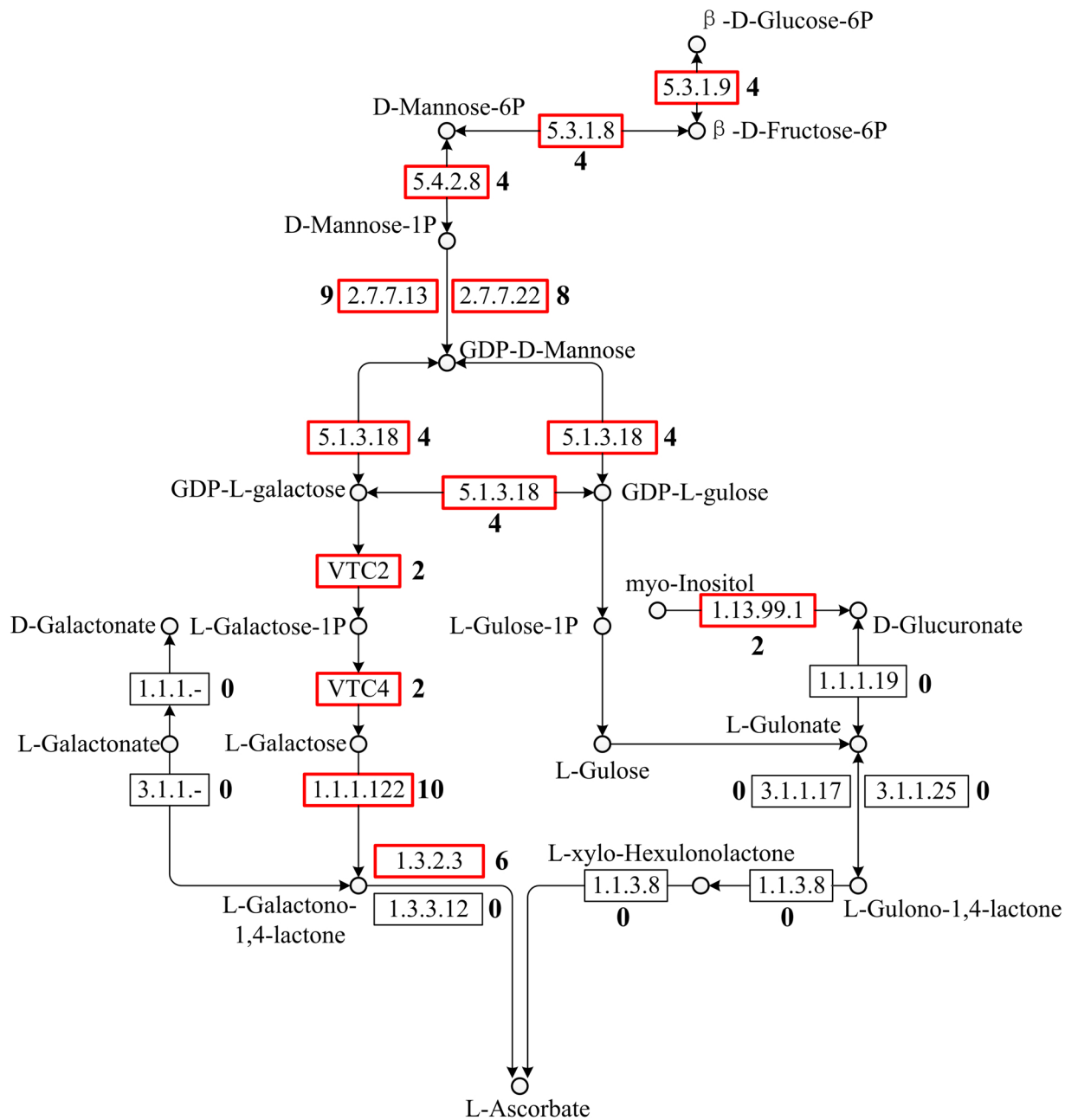

Supplement: Additional File 5 — Ascorbic acid biosynthesis pathway in Chinese bayberry. The number of UniGenes is shown besides each step. [file 1471-2164-13-19-S5.PDF]

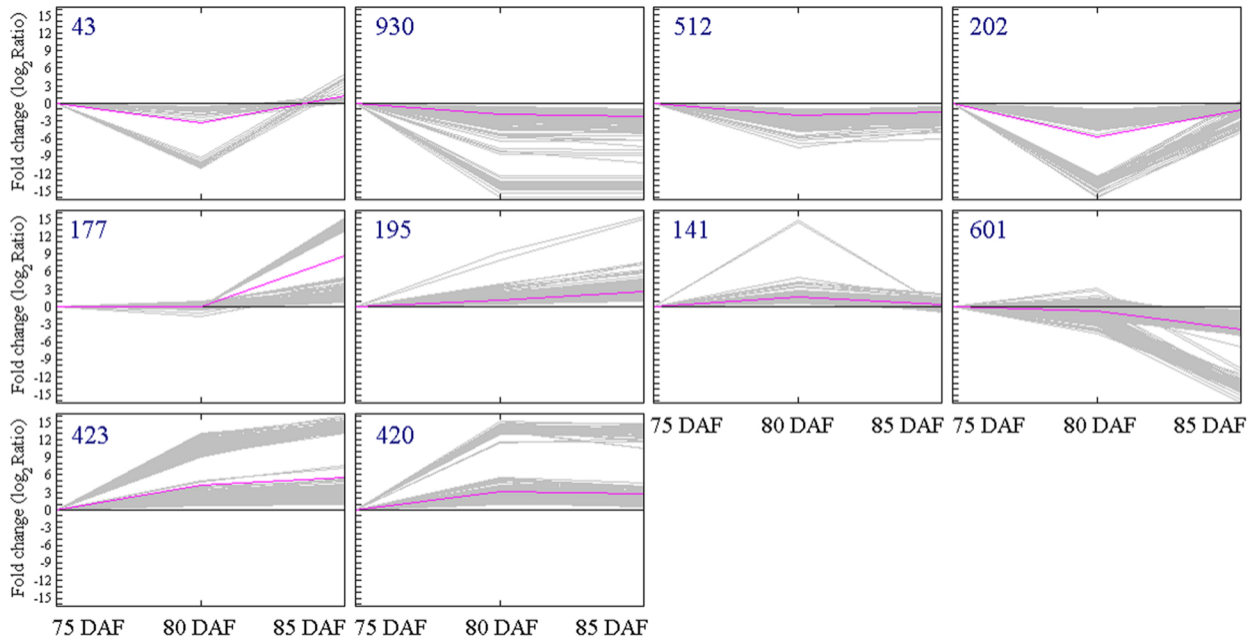

Supplement: Additional File 7 — Clustering analysis of differentially expressed UniGenes. [file 1471-2164-13-19-S7.PDF]

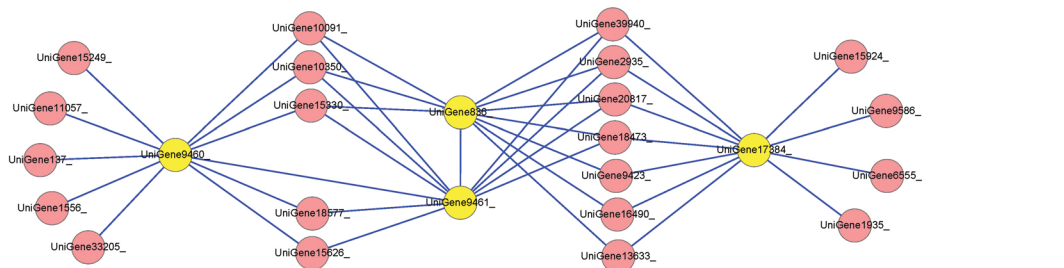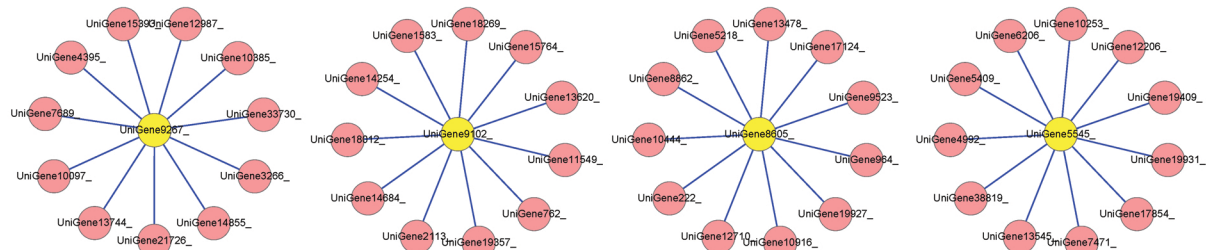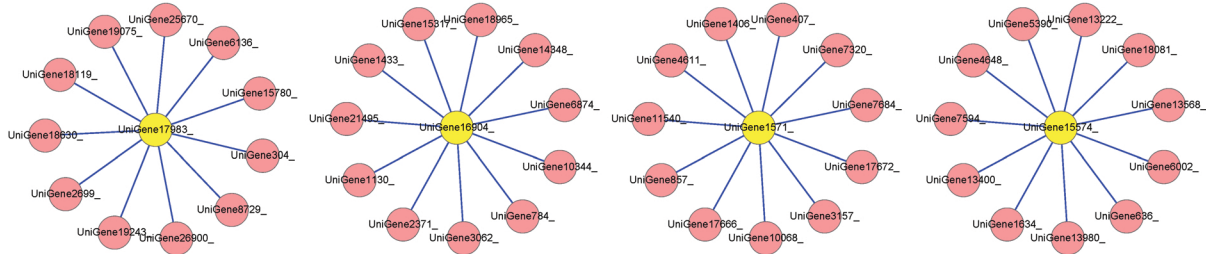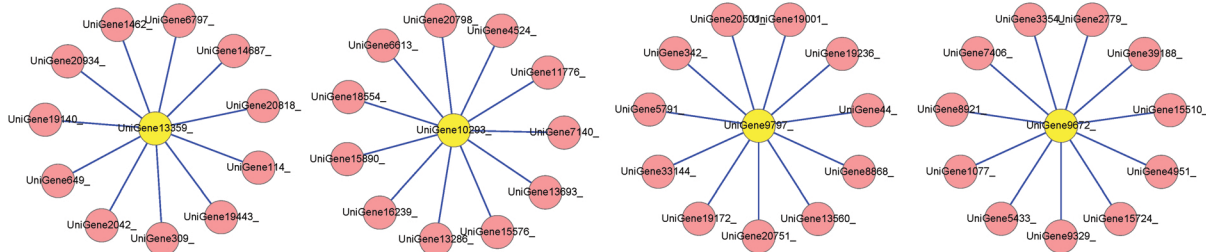

Supplement: Additional File 11 — The branch of co-expression network related with UniGenes in the anthocyanin biosynthesis pathway. The UniGenes encoding anthocyanin biosynthesis enzymes and related co-expressed UniGenes are indicated with yellow and pink red circles, respectively, and a line is drawn between co-expressed genes. [file 1471-2164-13-19-S11.PDF]
