# Supplementary material for: Transcriptomic analysis of Chinese bayberry (Myrica rubra) fruit development and ripening using RNA-Seq
Source: BMC Genomics. 2012 Jan 13;13:19. doi: 10.1186/1471-2164-13-19 (PMC3398333; doi:10.1186/1471-2164-13-19)
Supplement: Additional File 6 — COG classification of Chinese bayberry UniGenes. [file 1471-2164-13-19-S6.PDF]

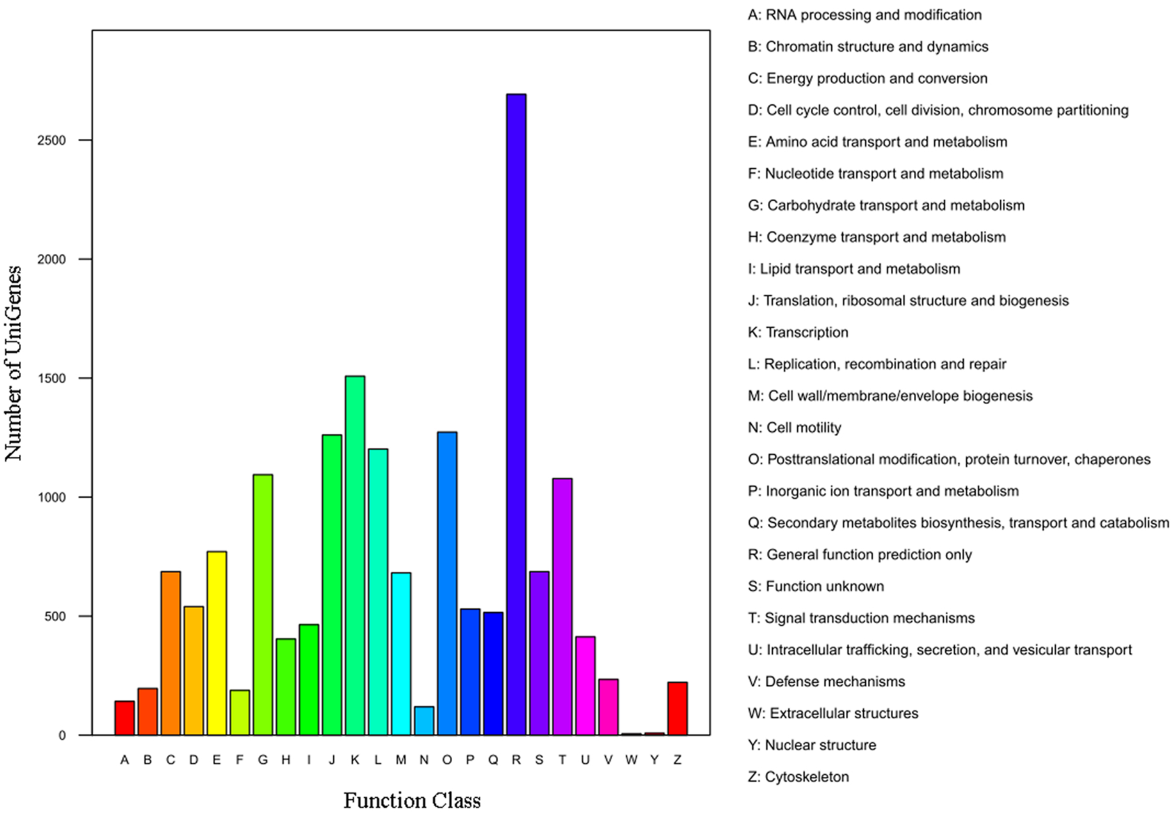

Number of UniGenes
0
500
1000
1500
2000
2500
A B C D E F G H I J K L M N O P Q R S T U V W Y Z
Function Class
A: RNA processing and modification
B: Chromatin structure and dynamics
C: Energy production and conversion
D: Cell cycle control, cell division, chromosome partitioning
E: Amino acid transport and metabolism
F: Nucleotide transport and metabolism
G: Carbohydrate transport and metabolism
H: Coenzyme transport and metabolism
I: Lipid transport and metabolism
J: Translation, ribosomal structure and biogenesis
K: Transcription
L: Replication, recombination and repair
M: Cell wall/membrane/envelope biogenesis
N: Cell motility
O: Posttranslational modification, protein turnover, chaperones
P: Inorganic ion transport and metabolism
Q: Secondary metabolites biosynthesis, transport and catabolism
R: General function prediction only
S: Function unknown
T: Signal transduction mechanisms
U: Intracellular trafficking, secretion, and vesicular transport
V: Defense mechanisms
W: Extracellular structures
Y: Nuclear structure
Z: Cytoskeleton
